# Fibrosis-Driven Cardiovascular Risk in Metabolic Dysfunction-Associated Steatotic Liver Disease: Emerging Links With Atherosclerotic Cardiovascular Disease

**DOI:** 10.7759/cureus.115501

**Published:** 2026-08-31

**Authors:** Nakul Mahajan, Nazdeep Kaur, Samar Pal S Sandhu, Vyoma Patel, Hunardeep Kaur Boparai, Kanwarmandeep Singh, FNU Rajinderpal Singh

**Affiliations:** 1 Internal Medicine, Maimonides Medical Center, New York City, USA; 2 Internal Medicine, Government Medical College, Amritsar, Amritsar, IND; 3 Internal Medicine, Icahn School of Medicine at Mount Sinai, Queens Hospital Center, New York City, USA

**Keywords:** atherosclerotic cardiovascular disease, cardio-hepatic axis, coronary artery calcium, endothelial dysfunction, fibrosis-4 index, glucagon-like peptide-1 receptor agonists, hepatic fibrosis, metabolic dysfunction-associated steatotic liver disease, subclinical atherosclerosis, transient elastography

## Abstract

Metabolic dysfunction-associated steatotic liver disease (MASLD) is increasingly recognized as a multisystem cardiometabolic disorder with major implications for atherosclerotic cardiovascular disease (ASCVD). Although hepatic steatosis has traditionally been considered the hallmark of MASLD, emerging evidence suggests that hepatic fibrosis severity rather than steatosis alone appears to be an important prognostic marker of adverse cardiovascular outcomes. These observations have prompted growing interest in incorporating fibrosis assessment into cardiovascular risk stratification and integrated cardio-hepatic care. This narrative review explores the evolving concept of the "fibrotic cardio-hepatic axis", highlighting the potential mechanistic pathways linking hepatic fibrosis with vascular injury and atherogenesis. Key mechanisms include chronic systemic inflammation, endothelial dysfunction, oxidative stress, proatherogenic lipid alterations, gut-liver-vascular interactions, and prothrombotic states. The review further discusses the expanding role of noninvasive fibrosis assessment tools, including the fibrosis-4 index, enhanced liver fibrosis score, and transient elastography, as emerging cardiovascular risk stratification instruments. Recent imaging studies linking fibrosis severity with coronary artery calcium progression, carotid atherosclerosis, coronary plaque burden, and myocardial remodeling are also examined. Additionally, contemporary therapeutic approaches including glucagon-like peptide-1 receptor agonists, sodium-glucose cotransporter-2 inhibitors, and novel anti-fibrotic agents are reviewed for their potential to simultaneously improve hepatic fibrosis and reduce ASCVD burden. Finally, future directions involving fibrosis-integrated cardiovascular risk models, precision medicine, artificial intelligence, and multidisciplinary cardio-hepatology care pathways are discussed. Recognition of fibrosis as an important cardiovascular risk marker may refine risk assessment and therapeutic strategies in MASLD.

## Introduction and background

Metabolic dysfunction-associated steatotic liver disease (MASLD), previously termed nonalcoholic fatty liver disease (NAFLD), has emerged as the hepatic manifestation of systemic metabolic dysfunction and is now recognized as a major contributor to cardiovascular morbidity and mortality. The recent transition from NAFLD to MASLD reflects an evolving understanding that hepatic steatosis is closely integrated with obesity, insulin resistance, type 2 diabetes mellitus, dyslipidemia, and chronic low-grade inflammation rather than being an isolated liver disorder [1]. Affecting nearly one-third of the global population, MASLD represents a substantial public health burden with implications extending far beyond progressive liver injury [2].

Atherosclerotic cardiovascular disease (ASCVD) has consistently remained the leading cause of death among patients with MASLD, surpassing liver-related complications in most longitudinal cohorts [3]. Earlier investigations primarily attributed this increased cardiovascular risk to shared metabolic comorbidities; however, accumulating evidence suggests that the severity of hepatic fibrosis itself independently predicts adverse cardiovascular outcomes [4]. This paradigm shift has redirected attention from simple steatosis toward fibrosis-associated systemic vascular injury and has introduced the concept of a "fibrotic cardio-hepatic axis", describing potential biological links between progressive hepatic fibrogenesis, inflammatory and metabolic alterations, and cardiovascular disease.

Importantly, cardiovascular risk appears heterogeneous across the histologic spectrum of MASLD. Patients with isolated steatosis often demonstrate comparatively modest cardiovascular risk, whereas those with steatohepatitis and advanced fibrosis exhibit significantly higher incidences of myocardial infarction, stroke, heart failure, and cardiovascular mortality [5]. Studies have reported that advanced fibrosis assessed using noninvasive fibrosis scores was independently associated with major adverse cardiovascular events (MACE) even after adjustment for traditional cardiovascular risk factors [6]. Similarly, liver stiffness measured through transient elastography has shown strong correlations with coronary artery calcification, carotid intima-media thickness, and subclinical atherosclerosis, further supporting an association between fibrosis severity and cardiovascular pathology [7].

The mechanistic basis underlying fibrosis-associated ASCVD is multifactorial. The proposed association involves interconnected inflammatory, metabolic, endothelial, and thrombotic pathways linking hepatic fibrosis with vascular injury [8]. Moreover, recent studies have demonstrated that fibrosis severity predicts incident heart failure and atrial fibrillation independent of obesity and diabetes status, emphasizing the systemic nature of fibrotic MASLD [9,10]. These findings challenge the traditional organ-specific perception of MASLD and support its recognition as a multisystem cardiometabolic disease.

Despite growing recognition of the association between hepatic fibrosis and cardiovascular disease in MASLD, the existing evidence remains fragmented across mechanistic, epidemiological, and clinical domains. In particular, uncertainty remains regarding the extent to which fibrosis severity contributes to cardiovascular risk beyond conventional metabolic risk factors, the utility of noninvasive fibrosis measures for cardiovascular risk stratification, and how these findings may be translated into integrated cardio-hepatic management. A comprehensive synthesis of these emerging data is therefore needed to clarify the clinical significance of fibrosis within the cardiovascular spectrum of MASLD.

Accordingly, this narrative review synthesizes the emerging evidence linking hepatic fibrosis with ASCVD, with particular emphasis on the mechanistic pathways underlying the cardio-hepatic axis, the role and limitations of noninvasive fibrosis assessment, associations with subclinical atherosclerosis and clinical cardiovascular outcomes, and evolving therapeutic implications for integrated cardiovascular and hepatic risk management.

## Review

Methodology

The present study was conducted as a structured narrative review to evaluate the association between MASLD, hepatic fibrosis, and ASCVD, with emphasis on proposed pathophysiological mechanisms, clinical evidence, noninvasive fibrosis assessment, and emerging therapeutic strategies. A structured literature search was performed in PubMed/MEDLINE, Scopus, the Cochrane Library, and Web of Science to identify relevant studies published in English from database inception through June 2026. Additional records were identified through manual screening of reference lists and citation searching to identify pertinent studies that were not captured through the database search. Search terms included combinations of "MASLD", "metabolic dysfunction-associated steatotic liver disease", "hepatic fibrosis", "atherosclerosis", "atherosclerotic cardiovascular disease", "coronary artery disease", "cardiovascular risk", "FIB-4", "transient elastography", and related keywords combined using the Boolean operators AND/OR. Database-specific adaptations of these terms were used according to the indexing requirements of each database. The search was limited to English-language publications, with no restriction on publication year.

Studies were considered eligible if they evaluated MASLD or NAFLD in relation to hepatic fibrosis, cardiovascular outcomes or subclinical cardiovascular disease, potential mechanistic links between hepatic fibrosis and cardiovascular disease, noninvasive assessment of hepatic fibrosis with potential implications for cardiovascular risk, or therapeutic strategies with relevant hepatic and/or cardiovascular effects. Randomized controlled trials, prospective and retrospective cohort studies, case-control studies, systematic reviews, meta-analyses, and major clinical practice guidelines were considered. Studies were excluded if they were unrelated to the review objectives, lacked clinically relevant hepatic or cardiovascular outcomes, consisted solely of experimental findings without sufficient relevance to the clinical context, or were conference abstracts, editorials, letters, or duplicate publications without substantive additional information.

The literature identification and selection process was documented using the Preferred Reporting Items for Systematic Reviews and Meta-Analyses (PRISMA) 2020 framework for transparency (Figure 1) [11]. However, the present study was not conducted as a systematic review or meta-analysis. A total of 1248 records were identified, including 1206 from electronic databases and 42 from other sources. After the removal of duplicates (n=287), records excluded by automation tools (n=13), and records removed for other reasons (n=12), 894 records underwent title and abstract screening, of which 742 were excluded. Full texts of 194 reports were assessed for eligibility (152 from the databases and 42 from websites and citation searching), and 106 were excluded because of insufficient data, lack of direct relevance to the review objectives, or inadequate hepatic or cardiovascular outcome information. Ultimately, 88 studies were included in the qualitative synthesis. A summary of the characteristics and principal findings of all 88 studies included in the qualitative synthesis is provided in the Appendices.

**Figure 1:**
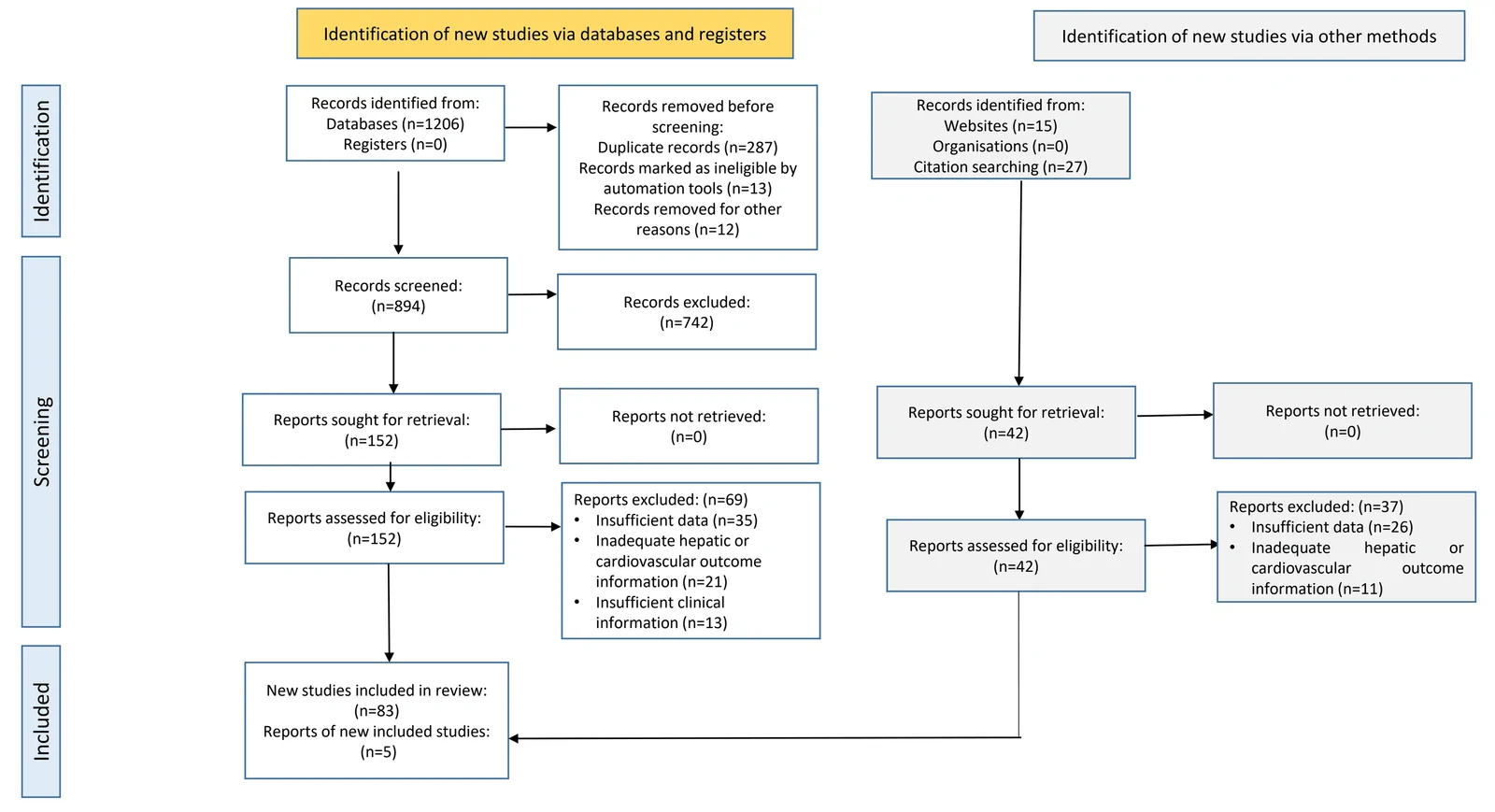
PRISMA flow diagram of the study selection process PRISMA: Preferred Reporting Items for Systematic Reviews and Meta-Analyses

Because this was a structured narrative review rather than a systematic review or meta-analysis, no formal study-level risk-of-bias assessment or quantitative data pooling was undertaken. Instead, the identified literature was critically synthesized according to study design, study population, fibrosis assessment method, cardiovascular outcomes, and relevance to the objectives of the review. Particular emphasis was placed on contemporary and clinically relevant evidence, while findings from observational studies were interpreted as associations and not as definitive evidence of causality. Mechanistic and experimental evidence was considered primarily to provide biological context for the observed clinical associations. The synthesis therefore focused on the emerging evidence linking hepatic fibrosis with cardiovascular risk while acknowledging the limitations of the predominantly observational clinical evidence.

Mechanistic pathways linking hepatic fibrosis to atherosclerosis

Chronic Systemic Inflammation in Fibrotic MASLD

Progressive hepatic fibrosis in MASLD is characterized by persistent low-grade systemic inflammation that may contribute to vascular injury and atherogenic processes. Fibrotic liver tissue demonstrates enhanced activation of Kupffer cells, hepatic stellate cells, and inflammasome signaling pathways, resulting in increased circulating levels of proinflammatory mediators including interleukin-6 (IL-6), tumor necrosis factor-alpha (TNF-α), and high-sensitivity C-reactive protein (hsCRP) [12]. These inflammatory cytokines promote endothelial activation, monocyte recruitment, and smooth muscle proliferation within the vascular wall, which may collectively contribute to vascular injury and plaque instability [13].

The NOD-, LRR-, and pyrin domain-containing protein 3 (NLRP3) inflammasome has emerged as a critical molecular link between hepatic fibrosis and ASCVD. Activation of inflammasome pathways promotes the secretion of IL-1β and IL-18, amplifying systemic vascular inflammation and endothelial injury [14]. Experimental studies have shown that fibrotic MASLD is associated with heightened inflammasome activity in both hepatic and vascular tissues, suggesting shared inflammatory signaling between the liver and arterial wall. Additionally, chronic inflammation associated with fibrosis contributes to macrophage polarization toward proatherogenic phenotypes, facilitating plaque progression and necrotic core formation [15].

Endothelial Dysfunction and Oxidative Stress

Endothelial dysfunction represents an early and pivotal step in fibrosis-associated atherosclerosis. Advanced hepatic fibrosis is associated with impaired nitric oxide bioavailability, increased reactive oxygen species (ROS) production, and vascular stiffness, all of which contribute to accelerated arterial aging [8]. Hepatic oxidative stress promotes systemic lipid peroxidation and mitochondrial dysfunction, resulting in endothelial injury and impaired vasodilation. A cross-sectional study of 7507 adults demonstrated that increasing MASLD severity was independently associated with greater coronary artery calcification, indicating a progressive relationship between disease severity and subclinical atherosclerosis beyond traditional cardiovascular risk factors [16].

Fibrotic MASLD is also associated with increased circulating asymmetric dimethylarginine (ADMA), an endogenous nitric oxide synthase inhibitor linked to impaired endothelial function and adverse cardiovascular outcomes [17]. Furthermore, ROS-mediated oxidation of low-density lipoproteins enhances foam cell formation and plaque vulnerability. Experimental models have demonstrated that transforming growth factor-beta (TGF-β), a key mediator of hepatic fibrogenesis, simultaneously promotes vascular fibrosis and arterial stiffness, reinforcing the concept of a shared fibrotic vascular phenotype [18].

Proatherogenic Lipoprotein and Metabolic Alterations

Advanced fibrosis in MASLD is associated with profound alterations in lipid metabolism that favor atherogenesis. Patients with fibrotic MASLD frequently exhibit elevated apolipoprotein B (ApoB), small dense low-density lipoprotein (sdLDL), remnant cholesterol, and severe insulin resistance [19]. These lipoprotein abnormalities are highly atherogenic due to their increased susceptibility to oxidation and enhanced arterial wall penetration. In a cross-sectional analysis of 2550 individuals with biopsy-confirmed MASLD, advanced fibrosis was independently associated with significantly higher ApoB levels and a 38% increase in coronary plaque burden on coronary computed tomography angiography [20].

Insulin resistance further amplifies cardiovascular risk through increased hepatic very-low-density lipoprotein secretion, impaired glucose utilization, and endothelial dysfunction. Importantly, recent metabolomic studies suggest that fibrosis severity correlates more strongly with proatherogenic lipid signatures than hepatic steatosis alone, supporting the concept that fibrosis represents a metabolically malignant phenotype within MASLD [21].

Gut-Liver-Vascular Axis

The gut-liver-vascular axis has recently gained significant attention as a mechanistic driver of fibrosis-associated ASCVD. Progressive fibrosis is associated with gut microbial dysbiosis, increased intestinal permeability, and systemic endotoxemia [22]. Translocation of bacterial endotoxins such as lipopolysaccharide activates toll-like receptor signaling, promoting hepatic inflammation and vascular endothelial injury. In a recent metagenomic study, patients with advanced MASLD fibrosis demonstrated significantly higher circulating endotoxin levels and increased prevalence of proatherogenic microbial species compared with individuals with isolated steatosis [23].

Trimethylamine N-oxide (TMAO), a gut-derived metabolite implicated in atherosclerosis, has also been linked with fibrosis severity. Elevated TMAO concentrations promote macrophage foam cell formation, platelet hyperreactivity, and endothelial dysfunction [24]. A comprehensive review highlighted that elevated TMAO is strongly associated with cardiovascular disease and is influenced by diet, gut microbial composition, hepatic flavin-containing monooxygenase 3 (FMO3) activity, and renal function [25]. These findings suggest that gut microbial alterations may represent a shared pathogenic pathway linking hepatic fibrosis with systemic vascular disease.

Prothrombotic and Plaque-Destabilizing State

Fibrotic MASLD is increasingly recognized as a prothrombotic condition characterized by platelet activation, coagulation imbalance, and vascular inflammation [26]. Advanced fibrosis is associated with elevated fibrinogen, factor VIII, plasminogen activator inhibitor-1 (PAI-1), and von Willebrand factor levels, all of which contribute to thrombogenesis and plaque instability [27]. Moreover, activated platelets release inflammatory mediators and microparticles that further exacerbate endothelial dysfunction and atherosclerotic progression.

In a longitudinal cohort study, patients with advanced fibrosis exhibited a significantly higher incidence of acute coronary syndromes and ischemic stroke compared with those without fibrosis, even after adjustment for metabolic risk factors [28]. Histopathologic analyses additionally demonstrate increased intraplaque inflammation and vascular macrophage infiltration among patients with fibrotic MASLD, supporting the hypothesis that hepatic fibrosis may be linked to processes involved in plaque formation, plaque destabilization, and thrombotic cardiovascular events [29]. The interconnected inflammatory, metabolic, endothelial, microbial, and thrombotic pathways linking hepatic fibrosis with vascular injury collectively constitute the proposed fibrotic cardio-hepatic axis in MASLD (Figure 2).

**Figure 2:**
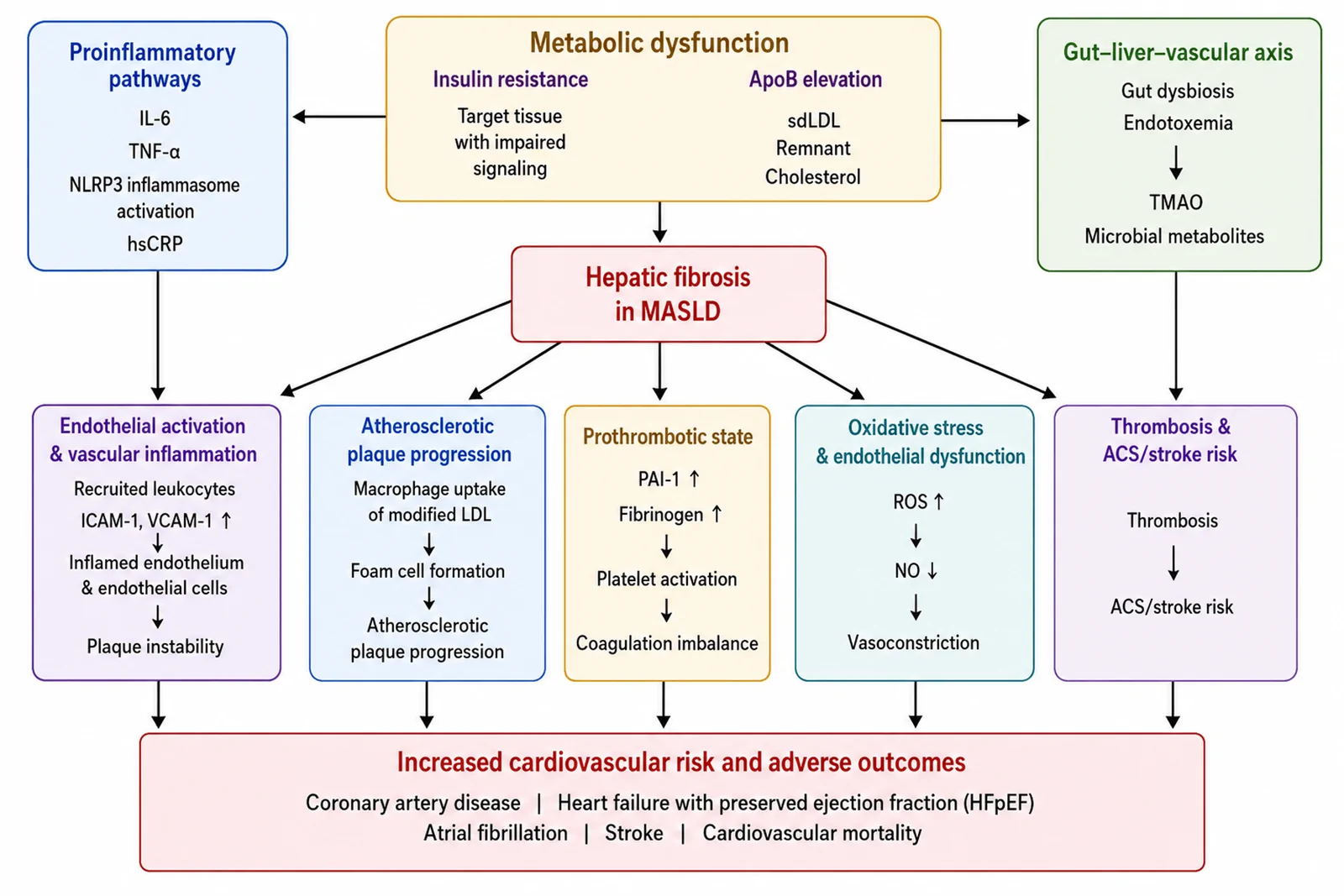
The fibrotic cardio-hepatic axis in MASLD Progressive hepatic fibrosis is associated with systemic inflammation, endothelial dysfunction, oxidative stress, gut microbial dysbiosis, proatherogenic lipid alterations, and a prothrombotic milieu, which may collectively contribute to atherosclerotic and adverse cardiovascular processes. ACS: acute coronary syndrome; ApoB: apolipoprotein B; hsCRP: high-sensitivity C-reactive protein; ICAM-1: intercellular adhesion molecule-1; IL-6: interleukin-6; LDL: low-density lipoprotein; MASLD: metabolic dysfunction-associated steatotic liver disease; NLRP3: NOD-, LRR-, and pyrin domain-containing protein 3; NO: nitric oxide; PAI-1: plasminogen activator inhibitor-1; ROS: reactive oxygen species; sdLDL: small dense low-density lipoprotein; TMAO: trimethylamine N-oxide; TNF-α: tumor necrosis factor-alpha; VCAM-1: vascular cell adhesion molecule-1 The figure was created by Kanwarmandeep Singh using Microsoft PowerPoint (Microsoft Corp., Redmond, WA, USA).

Noninvasive fibrosis assessment and detection of subclinical ASCVD

Fibrosis Scores as Cardiovascular Risk Predictors

The growing recognition of hepatic fibrosis as an important prognostic marker of cardiovascular risk in MASLD has intensified interest in noninvasive fibrosis assessment tools as predictors of ASCVD. Among these, the fibrosis-4 (FIB-4) index has emerged as one of the most extensively validated markers linking hepatic fibrosis with MACE, cardiovascular mortality, and subclinical coronary disease [30]. A retrospective cohort study of 19,395 patients with metabolic dysfunction-associated steatohepatitis (MASH) demonstrated that a high baseline FIB-4 score was independently associated with increased risks of major adverse liver outcomes (aHR: 4.29; 95% CI: 3.87-4.77) and MACE (aHR: 1.44; 95% CI: 1.25-1.66), while progression to a high-risk FIB-4 category further increased the risk of adverse liver outcomes (aHR: 2.62; 95% CI: 1.92-3.58) [31].

Similarly, the NAFLD fibrosis score (NFS) has shown strong associations with coronary artery disease (CAD) and adverse cardiovascular outcomes independent of traditional cardiometabolic risk factors [32]. In a prospective Korean cohort study, elevated NFS independently predicted incident ischemic stroke and coronary artery calcification progression, even after adjustment for diabetes, obesity, and hypertension [33]. The enhanced liver fibrosis (ELF) score has also gained attention due to its ability to directly reflect extracellular matrix turnover and active fibrogenesis. In a recent European study involving biopsy-confirmed MASLD, higher ELF scores were associated with significantly increased cardiovascular mortality and heart failure hospitalization rates, suggesting that active fibrotic remodeling may parallel systemic vascular injury [34].

Transient elastography, which quantifies liver stiffness noninvasively, has further strengthened the clinical connection between fibrosis and ASCVD. Elevated liver stiffness measurements have consistently correlated with CAD severity, vascular stiffness, and incident cardiovascular events [35]. It has been reported that patients with liver stiffness measurement ≥12 kPa demonstrated nearly a threefold increase in coronary plaque burden and significantly higher coronary artery calcium (CAC) scores compared with patients without advanced fibrosis [36]. These findings collectively suggest that fibrosis biomarkers may serve as clinically useful cardiovascular risk stratification tools in MASLD populations. More recent longitudinal evidence has further strengthened the prognostic utility of fibrosis biomarkers in MASLD. In a large nationwide cohort involving 134,686 individuals with MASLD, Lee and Lee demonstrated that higher FIB-4 scores were independently associated with a 48.9% increase in all-cause mortality risk and a 47.8% increase in cardiovascular mortality risk [37]. Notably, the association was more pronounced among younger individuals, suggesting that fibrosis-related cardiovascular risk may emerge earlier than traditionally appreciated. Similarly, dynamic changes in fibrosis biomarkers appear clinically relevant. A recent longitudinal study reported that persistently elevated or rising FIB-4 trajectories were independently associated with progressive CAC accumulation, particularly among individuals older than 40 years with MASLD [38]. These findings support the concept that serial fibrosis assessment may provide incremental value beyond static single-time-point measurements for cardiovascular risk prediction.

Imaging Evidence of Subclinical Atherosclerosis

Advanced fibrosis in MASLD is increasingly associated with imaging markers of subclinical atherosclerosis, supporting the concept that vascular injury begins before overt cardiovascular events become clinically apparent. Recent evidence additionally suggests that changes in liver fibrosis measures may be associated with changes in subclinical atherosclerosis burden, further supporting an association between fibrosis severity and vascular disease progression [20]. CAC scoring has emerged as a particularly valuable imaging modality in this context. Multiple studies have demonstrated that increasing fibrosis severity is independently associated with higher CAC progression and greater plaque burden [39]. In a cohort of 1173 asymptomatic adults, CAC progression occurred in 20.5% of participants without NAFLD, 27.5% with NAFLD and low FIB-4, and 35.9% with NAFLD and intermediate/high FIB-4, with the latter group showing greater adjusted odds of progression than participants without NAFLD (OR: 1.70; 95% CI: 1.12-2.58) [40].

Carotid intima-media thickness, a well-established marker of early atherosclerosis, also correlates strongly with fibrosis severity. Patients with advanced fibrosis exhibit significantly higher carotid intima-media thickness values and increased carotid plaque prevalence compared with those with isolated steatosis [41]. In a meta-analysis comprising over 12,000 MASLD patients, fibrosis severity demonstrated a stepwise association with subclinical carotid atherosclerosis independent of conventional cardiovascular risk factors [42].

Coronary computed tomography angiography studies have further revealed that fibrotic MASLD is associated with high-risk plaque phenotypes characterized by positive remodeling, low-attenuation plaques, and increased noncalcified plaque burden [43]. Additionally, epicardial adipose tissue, an inflammatory visceral fat depot closely linked with coronary atherosclerosis, is significantly increased in advanced fibrosis and correlates with both liver stiffness and vascular inflammation [44]. These imaging findings support an association between hepatic fibrosis and a systemic proatherogenic phenotype.

Fibrosis Severity and Cardiovascular Phenotypes

Emerging evidence suggests that fibrosis severity influences not only generalized ASCVD risk but also distinct cardiovascular phenotypes including CAD, heart failure with preserved ejection fraction (HFpEF), atrial fibrillation, and vascular stiffness. In a nationwide cohort study involving more than 10,000 patients with MASLD, advanced fibrosis independently predicted incident CAD with adjusted HRs exceeding 2.0 after controlling for metabolic risk factors [45]. Furthermore, fibrosis severity correlated directly with coronary plaque complexity and multivessel disease burden.

Additional evidence supporting the cardiovascular burden of MASLD comes from a multicenter propensity-matched longitudinal analysis of 48,552 patients derived from the TriNetX electronic health record network [46]. Compared with matched controls, patients with MASLD experienced a significantly higher five-year incidence of MACE, including myocardial infarction, stroke, and cardiovascular death. Notably, cardiovascular event curves diverged within 24 months, while statin therapy was associated with a significant reduction in cardiovascular risk, underscoring the importance of early cardiovascular risk assessment and preventive management in patients with MASLD.

HFpEF has also emerged as an important cardiovascular manifestation of fibrotic MASLD. Chronic systemic inflammation, myocardial fibrosis, endothelial dysfunction, and ventricular stiffness associated with hepatic fibrosis may collectively promote diastolic dysfunction [47]. In a recent cardiac magnetic resonance imaging study, patients with advanced MASLD fibrosis demonstrated significantly higher myocardial extracellular volume fractions and impaired diastolic strain parameters compared with controls, suggesting diffuse myocardial remodeling [48].

Similarly, fibrosis-associated atrial remodeling may increase susceptibility to atrial fibrillation. A large population-based analysis reported that patients with elevated FIB-4 scores had a 1.8-fold increased risk of incident atrial fibrillation independent of obesity and diabetes [49]. Vascular stiffness, another hallmark of fibrotic MASLD, has consistently correlated with liver stiffness measurements and endothelial dysfunction biomarkers [50]. Collectively, these observations reinforce the concept that hepatic fibrosis represents a systemic fibro-inflammatory state capable of influencing multiple cardiovascular phenotypes beyond traditional atherosclerosis alone. Beyond coronary and cardiac phenotypes, recent studies have also linked fibrosis severity with peripheral vascular disease manifestations. In a 2025 cross-sectional study involving 481 patients with MASLD, higher FIB-4 categories were independently associated with lower extremity arterial disease after adjustment for metabolic and lipid-related variables [51]. Additionally, fibrosis-integrated inflammatory markers such as systemic inflammation response index and neutrophil-to-lymphocyte ratio have demonstrated improved prediction of cardiovascular mortality when combined with FIB-4, highlighting the growing potential for multidimensional fibrosis-based cardiovascular risk models [52]. Collectively, these studies demonstrate that hepatic fibrosis severity consistently predicts subclinical atherosclerosis, adverse cardiovascular phenotypes, and major cardiovascular events across diverse MASLD populations (Table 1).

**Table 1:** Recent clinical studies linking hepatic fibrosis with ASCVD outcomes in MASLD ASCVD: atherosclerotic cardiovascular disease; CAC: coronary artery calcium; ELF: enhanced liver fibrosis; FIB-4: fibrosis-4 index; HFpEF: heart failure with preserved ejection fraction; aHR: adjusted hazard ratio; MASLD: metabolic dysfunction-associated steatotic liver disease; MACE: major adverse cardiovascular events; NFS: nonalcoholic fatty liver disease fibrosis score; MASH: metabolic dysfunction-associated steatohepatitis; NHANES: National Health and Nutrition Examination Survey; LSM: liver stiffness measurement; MI: myocardial infarction; Lp-PLA2: lipoprotein-associated phospholipase A2

Study	Year	Study design/population	Fibrosis assessment method	Cardiovascular outcome	Key findings
Gbadamosi et al. [31]	2025	Retrospective cohort; 19,395 patients with MASH	FIB-4 index	MACE, major adverse liver outcomes	High baseline FIB-4 independently predicted MACE (aHR: 1.44; 95% CI: 1.25-1.66) and liver events (aHR: 4.29; 95% CI: 3.87-4.77); rising FIB-4 further increased liver event risk
Lee and Lee [37]	2025	Nationwide cohort; n=134,686 MASLD patients	FIB-4 index	All-cause mortality, cardiovascular mortality	Higher FIB-4 independently associated with 48.9% higher all-cause mortality and 47.8% higher cardiovascular mortality
Suresh et al. [32]	2026	State-of-the-art review	FIB-4, NFS, and other fibrosis biomarkers	Incident ASCVD	Advanced fibrosis biomarkers consistently predicted cardiovascular disease independent of conventional risk factors
Oh et al. [33]	2021	Prospective Korean cohort (16-year follow-up)	Liver fibrosis indices (including NFS)	Mortality, cardiovascular outcomes	Elevated liver fibrosis indices independently predicted long-term mortality, supporting their prognostic value in cardiovascular risk assessment
Li et al. [34]	2026	Comprehensive review	ELF score	Cardiovascular mortality, heart failure hospitalization	Higher ELF scores associated with increased cardiovascular mortality and heart failure hospitalization
Sukocheva et al. [35]	2025	Review	Transient elastography (LSM)	ASCVD risk	Elevated liver stiffness was consistently associated with increased cardiovascular risk and vascular stiffness
Lee and Lee [38]	2025	Longitudinal cohort	Serial FIB-4 trajectories	CAC progression	Persistently elevated or rising FIB-4 independently predicted progressive CAC accumulation
Lee et al. [40]	2020	Prospective cohort	Noninvasive fibrosis assessment	Coronary artery calcification progression	Advanced liver fibrosis independently predicted accelerated CAC progression
Tsai et al. [39]	2022	Longitudinal imaging cohort	Liver fibrosis biomarkers	Coronary plaque progression	Liver fibrosis biomarkers independently predicted coronary plaque progression even in patients with low CAC scores
Ye et al. [41]	2025	Cross-sectional study	Fibrosis severity	Carotid atherosclerosis	Greater fibrosis severity increased the predictive value of Lp-PLA2 for carotid atherosclerosis
Jamalinia et al. [42]	2024	Systematic review and meta-analysis (>12,000 MASLD patients)	Multiple fibrosis assessment methods	Subclinical carotid atherosclerosis	Fibrosis severity demonstrated a stepwise association with carotid atherosclerosis independent of conventional cardiovascular risk factors
Jeong et al. [43]	2025	Observational cohort	Steatosis-associated fibrosis estimator	Subclinical coronary atherosclerosis	MASLD and fibrosis severity were independently associated with subclinical coronary atherosclerosis
Cernea et al. [44]	2025	Cross-sectional study	Liver fibrosis assessment	Epicardial adipose tissue	Advanced fibrosis correlated with increased epicardial adipose tissue and adverse cardiometabolic phenotype
Lee et al. [45]	2025	Nationwide cohort	MASLD severity	Cardiovascular risk	Increasing MASLD severity and cardiometabolic risk factor burden significantly increased cardiovascular risk
Salem et al. [46]	2025	Multicenter propensity-matched cohort; n=48,552	MASLD diagnosis	MACE, MI, stroke, cardiovascular death	MASLD was associated with higher 5-year MACE, MI (aHR: 1.56), stroke (aHR: 1.51), and cardiovascular death (aHR: 1.88); statin therapy reduced cardiovascular risk
Brar et al. [47]	2026	State-of-the-art review	Hepatic fibrosis	HFpEF	Fibrotic MASLD promotes HFpEF through systemic inflammation, myocardial fibrosis, and endothelial dysfunction
Nabilou et al. [48]	2026	Cardiac MRI study	Advanced MASLD fibrosis	Myocardial remodeling	Advanced fibrosis was associated with increased myocardial extracellular volume and impaired diastolic function
Yang et al. [49]	2026	Population-based cohort	Liver fibrosis burden	Incident arrhythmia	Higher liver fibrosis burden independently increased the risk of incident atrial fibrillation
Li et al. [50]	2025	NHANES analysis	MASLD-associated arterial stiffness	Arterial stiffness	MASLD was independently associated with increased arterial stiffness in young adults
Zhang et al. [51]	2025	Cross-sectional study; n=481 MASLD patients	FIB-4 categories	Lower extremity arterial disease	Higher FIB-4 categories independently predicted lower extremity arterial disease
Zhang et al. [52]	2025	Cardiovascular mortality analysis	FIB-4 plus inflammatory indices	Cardiovascular mortality	Combining FIB-4 with systemic inflammatory markers improved the prediction of cardiovascular mortality

Therapeutic implications: can treating fibrosis reduce ASCVD risk?

*Glucagon-Like Peptide-1 Receptor Agonists* (*GLP-1RAs)*

GLP-1RAs have emerged as promising therapeutic agents capable of simultaneously targeting hepatic fibrosis and ASCVD risk in MASLD. Beyond their glucose-lowering effects, GLP-1RAs exert anti-inflammatory, antiatherogenic, and anti-fibrotic actions that may interrupt the fibrotic cardio-hepatic axis [53]. Semaglutide has demonstrated significant histologic benefits in patients with MASH. In a phase 2 trial involving 320 patients with biopsy-confirmed MASH, semaglutide achieved MASH resolution without worsening fibrosis in 59% of patients compared with 17% in the placebo group after 72 weeks [54]. Although fibrosis improvement was modest, semaglutide significantly reduced inflammatory activity and cardiometabolic risk factors associated with vascular injury.

More recently, the ESSENCE phase 3 trial demonstrated that semaglutide resulted in clinically meaningful reductions in liver stiffness, body weight, hsCRP, and atherogenic lipid parameters, suggesting potential cardiovascular benefit through the attenuation of systemic inflammation and endothelial dysfunction [55]. Tirzepatide, a dual glucose-dependent insulinotropic polypeptide (GIP)/GLP-1RA, has shown even greater metabolic and hepatic efficacy. In the SYNERGY-NASH trial, tirzepatide achieved MASH resolution rates approaching 74% with fibrosis improvement in up to 51% of participants after 52 weeks [56]. Whether fibrosis improvement with tirzepatide translates into long-term ASCVD risk reduction remains to be established. Importantly, GLP-1RAs have already demonstrated robust cardiovascular protection in large cardiovascular outcome trials, including reductions in myocardial infarction, stroke, and cardiovascular mortality [57].

Sodium-Glucose Cotransporter-2 (SGLT2) Inhibitors

SGLT2 inhibitors have also gained substantial attention for their pleiotropic hepatic and cardiovascular benefits in MASLD. Experimental and clinical studies indicate that SGLT2 inhibitors reduce hepatic steatosis, improve insulin resistance, attenuate oxidative stress, and suppress inflammatory signaling pathways implicated in fibrogenesis [58]. In a multicenter prospective study, empagliflozin therapy for 48 weeks significantly reduced liver stiffness measurements and improved fibrosis biomarkers alongside reductions in hsCRP and vascular stiffness indices [59].

Cardiovascular benefits associated with SGLT2 inhibitors appear particularly relevant in fibrotic MASLD due to their favorable effects on endothelial function, myocardial energetics, and systemic inflammation. In patients with MASLD and type 2 diabetes mellitus, dapagliflozin therapy was associated with reductions in epicardial adipose tissue volume and improvements in coronary flow reserve, supporting a direct antiatherogenic effect [60]. Additionally, SGLT2 inhibitors consistently reduce hospitalization for heart failure and cardiovascular mortality across diverse cardiometabolic populations [61]. Given the strong association between hepatic fibrosis and HFpEF, these agents may provide dual cardio-hepatic protection by simultaneously mitigating myocardial and hepatic fibrosis.

Emerging Anti-fibrotic Therapies

Recent advances in anti-fibrotic pharmacotherapy have introduced novel agents specifically targeting pathways involved in hepatic fibrogenesis and systemic metabolic dysfunction. Resmetirom, a selective thyroid hormone receptor-β agonist, became the first therapy approved for MASH with fibrosis after demonstrating significant reductions in liver fibrosis and inflammatory activity [62]. In the MAESTRO-NASH trial, fibrosis improvement by at least one stage without worsening steatohepatitis occurred in 24-26% of patients receiving resmetirom compared with 14% in the placebo group [63]. Importantly, resmetirom also reduced low-density lipoprotein cholesterol, ApoB, and triglyceride levels, suggesting potential ASCVD risk reduction beyond hepatic benefits.

Lanifibranor, a pan-peroxisome proliferator-activated receptor agonist, has shown promising effects on fibrosis regression and vascular inflammation. In the NATIVE trial, lanifibranor achieved significant fibrosis improvement and reductions in inflammatory biomarkers linked with endothelial dysfunction [64]. Similarly, survodutide, a dual glucagon/GLP-1RA, demonstrated marked reductions in liver fat content, body weight, and fibrosis biomarkers in phase 2 studies [65].

Emerging incretin-based therapies such as pemvidutide and retatrutide may further redefine treatment paradigms in fibrotic MASLD. Pemvidutide has shown substantial reductions in hepatic fat fraction and cardiometabolic risk markers including triglycerides and hsCRP [66]. Retatrutide, a triple agonist targeting GLP-1, GIP, and glucagon receptors, has demonstrated profound weight reduction exceeding 24% alongside improvements in insulin resistance and inflammatory biomarkers associated with vascular dysfunction [67]. Although long-term cardiovascular outcome data remain limited, these agents may ultimately offer integrated treatment strategies capable of simultaneously attenuating hepatic fibrosis and ASCVD burden.

Lifestyle Interventions and Fibrosis Regression

Lifestyle modification remains the cornerstone of MASLD management and continues to provide the strongest evidence for simultaneous hepatic and cardiovascular benefit. Sustained weight reduction is closely associated with fibrosis regression and improved vascular outcomes. A prospective study involving serial liver biopsies demonstrated that weight loss ≥10% resulted in fibrosis regression in nearly 45% of patients and complete resolution of steatohepatitis in a substantial proportion [68]. Even moderate weight reduction of 7-10% has been associated with improvements in endothelial function, arterial stiffness, and inflammatory biomarkers linked to ASCVD [69].

The Mediterranean diet has consistently demonstrated favorable effects on both hepatic fibrosis and cardiovascular health. Rich in monounsaturated fats, polyphenols, and anti-inflammatory nutrients, the Mediterranean dietary pattern reduces oxidative stress and improves insulin sensitivity [70]. In randomized clinical trials, adherence to a Mediterranean diet significantly reduced liver stiffness measurements and carotid intima-media thickness while improving lipid profiles and endothelial function [71].

Exercise independently contributes to fibrosis regression and vascular protection through improvements in mitochondrial function, nitric oxide bioavailability, and systemic inflammation. Aerobic and resistance exercise interventions have demonstrated reductions in hepatic fat content, vascular stiffness, and epicardial adipose tissue even in the absence of significant weight loss [72]. These findings suggest that interventions targeting metabolic dysfunction and fibrosis may provide complementary hepatic and cardiovascular benefits (Table 2).

**Table 2:** Emerging therapeutic strategies targeting the fibrotic cardio-hepatic axis in MASLD ApoB: apolipoprotein B; ASCVD: atherosclerotic cardiovascular disease; GIP: glucose-dependent insulinotropic polypeptide; GLP-1: glucagon-like peptide-1; hsCRP: high-sensitivity C-reactive protein; LDL-C: low-density lipoprotein cholesterol; MASH: metabolic dysfunction-associated steatohepatitis; MASLD: metabolic dysfunction-associated steatotic liver disease; PPAR: peroxisome proliferator-activated receptor; SGLT2: sodium-glucose cotransporter-2

Therapeutic category	Therapy	Mechanism of action	Effects on hepatic fibrosis	Cardiovascular/vascular effects	Key study(s)
Incretin-based therapies	Semaglutide	GLP-1 receptor agonist; improves insulin sensitivity and suppresses inflammatory signaling	Promotes MASH resolution and reduces liver stiffness	Reduces hsCRP, body weight, atherogenic lipids, and major cardiovascular events	Newsome et al. [54]; ESSENCE trial [55]
Incretin-based therapies	Tirzepatide	Dual GIP/GLP-1 receptor agonist	Significant fibrosis improvement and MASH resolution	Reduces ApoB, triglycerides, systemic inflammation, and cardiometabolic risk	SYNERGY-NASH trial [56]
SGLT2 inhibitors	Empagliflozin	SGLT2 inhibition causing glucosuria and metabolic unloading	Improves fibrosis biomarkers and liver stiffness	Improves endothelial function, vascular stiffness, and heart failure outcomes	Multicenter prospective study [59]
SGLT2 inhibitors	Dapagliflozin	SGLT2 inhibitor with anti-inflammatory and metabolic effects	Reduces hepatic fat accumulation and fibrotic signaling	Reduces epicardial adipose tissue and improves coronary flow reserve	Clinical study [60]
Emerging anti-fibrotic agents	Resmetirom	Selective thyroid hormone receptor-β agonist	Histologic fibrosis regression and reduction in steatohepatitis activity	Lowers LDL-C, ApoB, triglycerides, and proatherogenic lipid burden	Harrison et al. [62]; Lurje and Tacke [63]
Emerging anti-fibrotic agents	Lanifibranor	Pan-PPAR agonist modulating lipid metabolism and inflammation	Improves fibrosis stage and inflammatory activity	Improves endothelial inflammatory profile and metabolic dysfunction	Francque et al. [64]
Emerging anti-fibrotic agents	Survodutide	Dual glucagon/GLP-1 receptor agonist	Reduces fibrosis biomarkers and liver fat content	Improves weight-related vascular and inflammatory parameters	Sanyal et al. [65]
Incretin-based therapies	Pemvidutide	GLP-1/glucagon dual receptor agonist	Reduces hepatic fat fraction and metabolic injury	Improves triglycerides, hsCRP, and systemic inflammatory burden	Browne et al. [66]
Incretin-based therapies	Retatrutide	Triple GLP-1/GIP/glucagon receptor agonist	Potential indirect fibrosis improvement through profound metabolic effects	Produces marked weight reduction and improves insulin resistance-associated vascular dysfunction	Jastreboff et al. [67]
Lifestyle interventions	Weight loss ≥10%	Reduces adipose inflammation and hepatic lipotoxicity	Strongly associated with fibrosis regression and MASH resolution	Improves systemic inflammation and ASCVD risk markers	Vilar-Gomez et al. [68]
Lifestyle interventions	Mediterranean diet	Anti-inflammatory dietary pattern rich in monounsaturated fats and antioxidants	Associated with reduced liver stiffness and fibrosis progression	Improves endothelial function, lipid profile, and carotid atherosclerosis markers	Estruch et al. [70]; Gepner et al. [71]
Lifestyle interventions	Aerobic exercise	Enhances mitochondrial oxidation and insulin sensitivity	Reduces hepatic steatosis and fibrosis-associated metabolic stress	Improves arterial stiffness and nitric oxide bioavailability	Keating et al. [69]
Lifestyle interventions	Resistance exercise	Improves skeletal muscle glucose utilization and metabolic homeostasis	Reduces hepatic fat independent of weight loss	Improves vascular function and reduces epicardial adipose tissue	Hallsworth et al. [72]

Future directions in fibrosis-based cardiovascular risk stratification

Should Fibrosis Scores Be Incorporated Into ASCVD Risk Models?

Conventional cardiovascular risk prediction tools, including the Framingham Risk Score and Systematic Coronary Risk Evaluation 2 (SCORE2), were developed primarily around established cardiovascular risk factors and do not specifically account for hepatic fibrosis or systemic fibro-inflammatory burden [73]. Consequently, they may not fully capture cardiovascular risk in patients with MASLD, particularly among those with advanced fibrosis. Emerging cohort data suggest that fibrosis-based markers may provide incremental prognostic information beyond conventional risk models [74]. Although much of this evidence is not specific to MASLD populations, it provides support for the broader prognostic relevance of fibrosis markers in cardiovascular disease. In a prospective cohort of 2726 participants with heart failure followed for nine years in the MyoVasc study, the FIB-4 index independently predicted all-cause mortality (HR: 1.341; 95% CI: 1.273-1.412; p<0.0001) and was associated with adverse cardiac functional and structural changes; notably, these associations were strongest in patients without MASLD [75].

Similarly, liver stiffness measured by transient elastography independently predicted cardiovascular mortality after adjustment for diabetes, hypertension, obesity, and dyslipidemia [76]. Although these findings do not establish that fibrosis scores improve ASCVD risk prediction specifically in MASLD, they suggest that hepatic fibrosis may represent a clinically relevant cardiovascular risk-enhancing characteristic beyond conventional risk factors. Further studies in well-characterized MASLD populations are therefore needed to determine whether incorporating fibrosis-based measures into established ASCVD risk models improves risk discrimination, calibration, and clinical decision-making.

Importantly, fibrosis severity may provide information that is not captured by hepatic steatosis alone. Emerging evidence suggests that cardiovascular risk may remain substantial in patients with relatively modest hepatic fat accumulation when fibrosis burden is high [77]. Nevertheless, whether fibrosis measures should be formally incorporated into established ASCVD risk calculators remains uncertain. Future studies should determine whether adding FIB-4, liver stiffness, or other validated fibrosis measures improves risk reclassification, clinical decision-making, and ultimately patient outcomes.

Multi-omics and Precision Medicine

The increasing availability of genomic, metabolomic, proteomic, and computational approaches may further refine fibrosis-based cardiovascular risk assessment. Genetic variants such as patatin-like phospholipase domain-containing protein 3 (PNPLA3) and transmembrane 6 superfamily member 2 (TM6SF2) are associated with hepatic lipid accumulation and fibrosis progression, while emerging evidence suggests that genetic determinants of liver disease may also intersect with systemic inflammatory and vascular pathways [78,79].

Metabolomic studies have similarly identified lipidomic and inflammatory signatures associated with both advanced fibrosis and coronary plaque burden. Associations involving branched-chain amino acids, ceramides, and oxidized phospholipids suggest that metabolic remodeling accompanying fibrosis may provide additional information regarding cardiovascular phenotype and disease burden [80]. These findings raise the possibility that future risk models could integrate clinical variables with fibrosis biomarkers, imaging, and molecular signatures rather than relying on a single fibrosis measure.

Artificial intelligence (AI) and machine-learning approaches may facilitate such multidimensional risk assessment. Models incorporating fibrosis biomarkers, imaging characteristics, genomic information, and conventional cardiovascular variables have demonstrated improved predictive performance compared with traditional risk calculators [81]. A retrospective cohort study of 2000 patients found that AI-based models, particularly deep neural networks, outperformed the Framingham Risk Score and ASCVD estimator for five-year cardiovascular event prediction, achieving an area under the curve (AUC) of 0.91 versus 0.76 and 0.74, respectively [82]. Although these findings are promising, external validation, calibration across diverse populations, and prospective assessment of clinical utility are required before such models can be incorporated into routine practice.

Cardio-Hepatology Clinics and Integrated Care

The overlapping metabolic, hepatic, and cardiovascular manifestations of MASLD provide a rationale for integrated models of care. Conventional organ-specific approaches may inadequately address the interaction between hepatic fibrosis, metabolic dysfunction, and cardiovascular disease, particularly in patients with advanced fibrosis and multiple cardiometabolic risk factors [83]. Multidisciplinary cardio-hepatology pathways involving hepatologists, cardiologists, endocrinologists, dietitians, and preventive-care specialists could facilitate coordinated assessment and management.

Integrated screening approaches combining fibrosis assessment with cardiovascular risk evaluation and metabolic profiling may improve the recognition of subclinical cardiovascular disease [84]. In a pilot multidisciplinary clinic study, combining liver stiffness assessment with cardiovascular imaging increased the detection of previously unrecognized high-risk cardiovascular phenotypes among patients with advanced fibrosis [85]. Such models may also facilitate coordinated use of therapies with complementary hepatic and cardiovascular effects, including GLP-1RAs and SGLT2 inhibitors.

Beyond diagnostic assessment, integrated care may improve the implementation of lifestyle interventions and cardiovascular prevention through coordinated patient education and longitudinal follow-up [86]. However, the clinical and cost-effectiveness of dedicated cardio-hepatology pathways remain to be established. Future studies should therefore evaluate whether integrated care models improve cardiovascular outcomes, treatment adherence, and healthcare utilization compared with conventional care.

Key Knowledge Gaps

Despite substantial progress in defining the relationship between hepatic fibrosis and cardiovascular disease, several important uncertainties remain. First, the extent to which fibrosis directly contributes to vascular disease versus serving as a marker of underlying systemic metabolic dysfunction has not been definitively established [87]. Although mechanistic evidence supports biological pathways linking hepatic fibrogenesis with vascular injury, much of the clinical evidence remains observational, limiting causal inference.

Second, fibrosis-focused randomized controlled trials evaluating hard cardiovascular outcomes remain limited. Most therapeutic studies have primarily assessed histologic improvement, liver stiffness, or metabolic surrogate outcomes rather than incident myocardial infarction, stroke, MACE, or cardiovascular mortality [88]. Consequently, improvement in hepatic fibrosis should not yet be assumed to translate directly into cardiovascular benefit.

Third, long-term cardiovascular outcome data for several emerging therapies remain incomplete. Although agents such as resmetirom, tirzepatide, and survodutide have demonstrated improvements in hepatic and cardiometabolic parameters, their effects on coronary plaque progression, vascular inflammation, and cardiovascular mortality require longer-term evaluation [89]. Similarly, clinically meaningful thresholds for fibrosis-based cardiovascular risk stratification and the optimal frequency of reassessment remain uncertain.

Future research should therefore prioritize prospective cardiovascular outcome studies, external validation of fibrosis-integrated ASCVD prediction models, standardized fibrosis thresholds for cardiovascular risk assessment, and mechanistic studies addressing the bidirectional relationship between hepatic fibrogenesis and vascular disease. Studies should also determine whether fibrosis-guided screening and treatment strategies improve patient-centered outcomes compared with conventional cardiometabolic risk assessment. Ultimately, an integrated approach combining noninvasive fibrosis assessment, selective evaluation of subclinical ASCVD, and coordinated multidisciplinary management may offer a practical framework for identifying and treating patients with MASLD who have the greatest cardiovascular risk (Figure 3).

**Figure 3:**
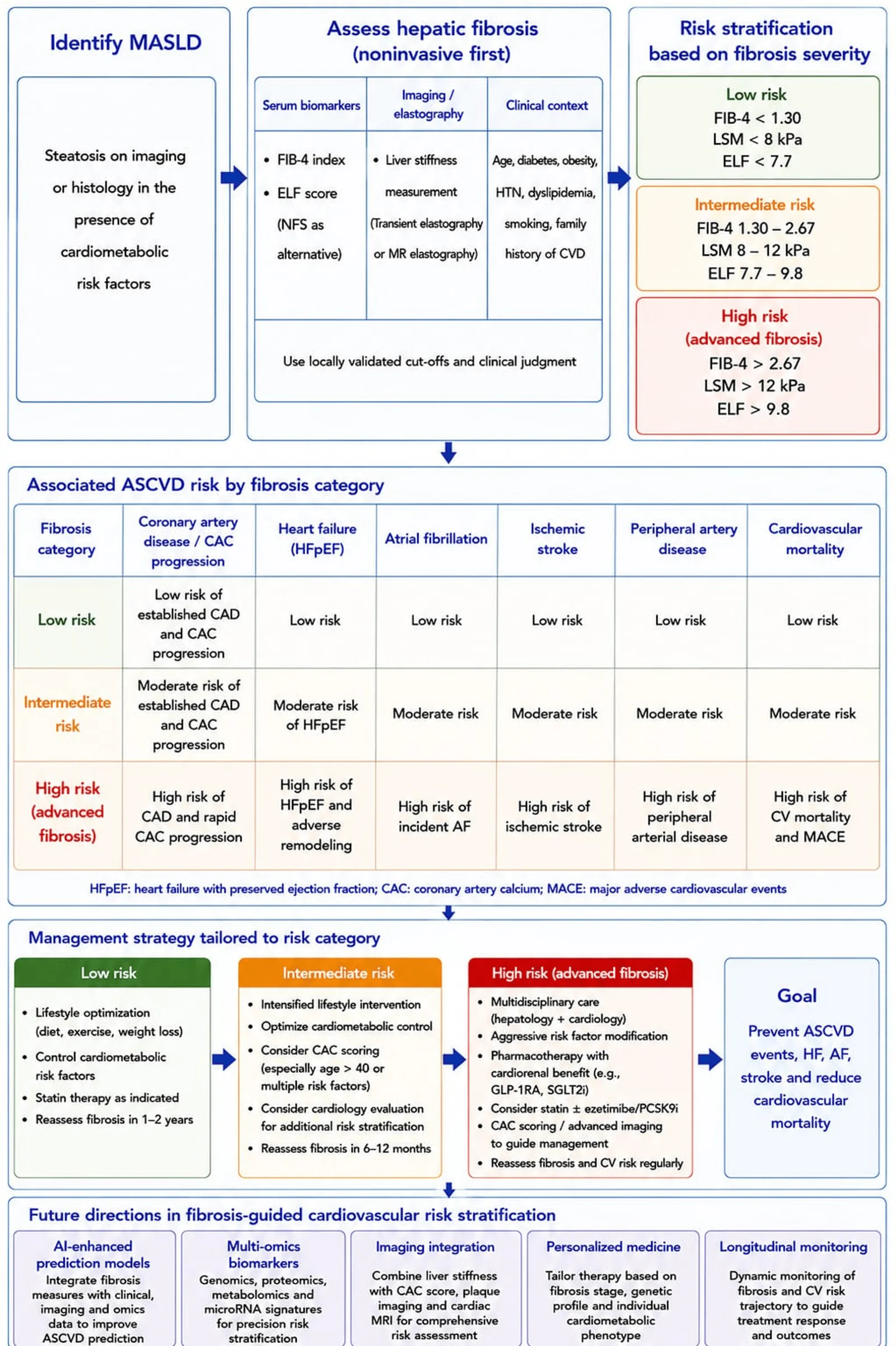
Clinical risk stratification algorithm for cardiovascular risk assessment in MASLD based on hepatic fibrosis severity AF: atrial fibrillation; ASCVD: atherosclerotic cardiovascular disease; CAC: coronary artery calcium; CVD: cardiovascular disease; ELF: enhanced liver fibrosis; FIB-4: fibrosis-4 index; GLP-1RA: glucagon-like peptide-1 receptor agonist; HF: heart failure; HFpEF: heart failure with preserved ejection fraction; HTN: hypertension; LSM: liver stiffness measurement; MACE: major adverse cardiovascular events; MASLD: metabolic dysfunction-associated steatotic liver disease; MR: magnetic resonance; MRI: magnetic resonance imaging; NFS: nonalcoholic fatty liver disease fibrosis score; PCSK9i: proprotein convertase subtilisin/kexin type 9 inhibitor; SGLT2i: sodium-glucose cotransporter-2 inhibitor; CAD: coronary artery disease; CV: cardiovascular The figure was created by Nakul Mahajan using Microsoft PowerPoint (Microsoft Corp., Redmond, WA, USA).

## Conclusions

Emerging evidence increasingly supports hepatic fibrosis as an important marker of cardiovascular risk in MASLD, extending the clinical significance of the disease beyond hepatic injury alone. Greater fibrosis severity is associated with systemic inflammation, endothelial dysfunction, subclinical atherosclerosis, and adverse cardiovascular phenotypes including CAD, heart failure, and atrial fibrillation. These observations support the concept of a fibrotic cardio-hepatic axis while acknowledging that the extent to which fibrosis itself contributes causally to cardiovascular disease remains incompletely established.

Noninvasive fibrosis assessment tools such as FIB-4, ELF score, and transient elastography may therefore have future utility in cardiovascular risk stratification among patients with MASLD. Emerging metabolic and anti-fibrotic therapies may simultaneously improve hepatic and cardiometabolic outcomes, although whether fibrosis improvement translates into fewer ASCVD events remains uncertain. Prospective fibrosis-focused cardiovascular outcome studies are needed to establish the clinical utility of fibrosis-based risk stratification and integrated cardio-hepatic management.

## Appendices

**Table 3:** Summary table of the 88 studies included in the review Note: Page et al. [11] describe the PRISMA 2020 statement, which provides methodological guidance for the reporting of systematic reviews. As this reference does not represent an original clinical, observational, or experimental study included in the qualitative synthesis, it has not been included. PRISMA: Preferred Reporting Items for Systematic Reviews and Meta-Analyses; NHANES: National Health and Nutrition Examination Survey; NAFLD: nonalcoholic fatty liver disease; MASLD: metabolic dysfunction-associated steatotic liver disease; MASH: metabolic dysfunction-associated steatohepatitis; CAD: coronary artery disease; RF: random forest; SVM: support vector machine; DNN: deep neural network; NLRP3: NOD-, LRR-, and pyrin domain-containing protein 3; TMAO: trimethylamine N-oxide; ASCVD: atherosclerotic cardiovascular disease; FIB-4: fibrosis-4 index; ELF: enhanced liver fibrosis; CAC: coronary artery calcium; HFpEF: heart failure with preserved ejection fraction; GLP-1: glucagon-like peptide-1; SGLT2: sodium-glucose cotransporter-2; PNPLA3: patatin-like phospholipase domain-containing protein 3; TM6SF2: transmembrane 6 superfamily member 2; FRS: Framingham Risk Score; NFS: NAFLD fibrosis score; Lp-PLA2: lipoprotein-associated phospholipase A2; ApoB: apolipoprotein B; LDL: low-density lipoprotein; hsCRP: high-sensitivity C-reactive protein; GIP: glucose-dependent insulinotropic polypeptide; EHR: electronic health records; AUC: area under the curve

First author [ref]	Year	Study design	Population/model	Study focus	Principal findings relevant to this review
Rinella [1]	2023	Multi-society Delphi consensus statement	International expert panel	MASLD nomenclature	Established the new MASLD nomenclature and diagnostic framework, emphasizing the metabolic basis of steatotic liver disease
Souza [2]	2025	Systematic review and meta-analysis	Patients with type 1 diabetes mellitus	Epidemiology of MASLD	Demonstrated a substantial global burden of MASLD in individuals with type 1 diabetes and summarized the associated clinical characteristics
Han [3]	2024	Nationwide population-based cohort	Patients with MASLD	Mortality outcomes	Reported that MASLD was associated with significantly increased all-cause and cardiovascular mortality
Lam [4]	2024	Retrospective cohort	Biopsy-proven MASLD	Advanced fibrosis	Demonstrated that advanced hepatic fibrosis independently predicted adverse liver outcomes and disease progression
Parikh [5]	2019	Observational cohort	Patients with NAFLD and advanced fibrosis	Stroke risk	Advanced liver fibrosis was independently associated with an increased risk of ischemic stroke
Liu [6]	2024	Population-based cohort	General adult population	Noninvasive fibrosis scores	Higher liver fibrosis scores independently predicted incident heart failure after adjustment for conventional cardiovascular risk factors
Denimal [7]	2025	Prospective cohort	Patients with type 2 diabetes and MASLD	Lower limb arterial calcification	MASLD biomarkers were associated with progression of lower extremity arterial calcification, supporting systemic vascular involvement
Kasper [8]	2021	Clinical review	Patients with NAFLD	Cardiovascular disease mechanisms	Reviewed the evidence linking NAFLD/MASLD with endothelial dysfunction, inflammation, atherosclerosis, and cardiovascular disease
Guo [9]	2024	Cohort study	Patients with heart failure	Liver fibrosis scores	Liver fibrosis scores independently predicted all-cause and cardiovascular mortality among patients with heart failure
Dawod [10]	2024	Narrative review	Patients with MASLD	Noninvasive fibrosis assessment	Summarized the clinical utility of noninvasive fibrosis tests including FIB-4, ELF score, and elastography for MASLD evaluation
National Institute of Diabetes and Digestive and Kidney Diseases [12]	2021	Government clinical guidance	General population	NAFLD/MASLD overview	Provided comprehensive clinical information regarding the epidemiology, diagnosis, complications, and management of fatty liver disease
Zhong [13]	2026	Original research	Patients with MASLD	Cardiovascular biomarkers	Identified the novel biomarkers useful for cardiovascular risk stratification in MASLD beyond conventional risk factors
Ma [14]	2025	Review	Experimental and clinical evidence	NLRP3 inflammasome	Described the role of NLRP3 inflammasome activation in hepatic inflammation, fibrosis, and systemic vascular injury
Francque [15]	2016	Comprehensive review	NAFLD populations	Pathophysiology	Summarized the inflammatory, metabolic, and endothelial mechanisms linking fatty liver disease with cardiovascular disease
Ma [16]	2025	Cross-sectional study	Adults with MASLD	Coronary atherosclerosis	Demonstrated an independent association between MASLD severity and subclinical coronary atherosclerosis
Zheng [17]	2024	Comprehensive review	MASLD populations	Cardiovascular risk	Reviewed the mechanisms through which MASLD promotes cardiovascular disease, including endothelial dysfunction, inflammation, and dyslipidemia
Wang [18]	2024	Review	Experimental studies	Transforming growth factor-β signaling	Discussed the central role of transforming growth factor-β signaling in hepatic fibrogenesis and vascular remodeling
Elsabaawy [19]	2025	Cross-sectional study	Patients with MASLD	Metabolic risk burden	Increasing metabolic risk factor burden was associated with progressively higher cardiovascular risk among patients with MASLD
Zhang [20]	2025	Cross-sectional and longitudinal study	Patients with MASLD	Liver fibrosis and subclinical atherosclerosis	Liver fibrosis independently increased the risk of subclinical atherosclerosis and vascular disease progression
Naja [21]	2025	Metabolomic study	Patients with MASLD	Metabolic signatures	Identified the metabolomic alterations associated with fibrosis progression and cardiometabolic dysfunction in MASLD
Tilg [22]	2021	Review	Experimental and clinical evidence	Gut-liver-immune axis	Highlighted the interactions among gut microbiota, metabolism, immunity, and liver disease that contribute to fibrosis progression and systemic inflammation
Satthawiwat [23]	2024	Cross-sectional microbiome study	Patients with MASLD	Gut microbiome and fibrosis	Identified the distinct gut microbial signatures and host genetic variants associated with advanced fibrosis, supporting the gut-liver-vascular axis in MASLD
Tang [24]	2017	Review	Experimental and clinical studies	TMAO	Summarized the role of TMAO as a gut-derived metabolite promoting endothelial dysfunction, foam cell formation, platelet activation, and atherosclerosis
Karaağaç [25]	2023	Narrative review	Clinical evidence	TMAO as cardiovascular biomarker	Reviewed the evidence supporting elevated circulating TMAO as an independent biomarker for cardiovascular disease influenced by diet, gut microbiota, hepatic metabolism, and renal function
Lombardi [26]	2025	Prospective observational study	Patients with MASLD	Procoagulant imbalance	Demonstrated that advanced hepatic fibrosis is associated with coagulation abnormalities contributing to hepatic and vascular complications
González-Mendoza [27]	2025	Review	Patients with MASLD	Hemostatic alterations	Summarized the evidence showing increased fibrinogen, factor VIII, plasminogen activator inhibitor-1, and other prothrombotic factors in MASLD
Baek [28]	2024	Nationwide cohort	Adults with MASLD	Cardiovascular outcomes	Liver fibrosis independently predicted incident coronary events and ischemic stroke after adjustment for metabolic risk factors
Kawanaka [29]	2025	Longitudinal cohort	Patients with MASLD	Predictors of ASCVD events	Demonstrated that advanced liver fibrosis significantly increased future ASCVD events beyond conventional cardiovascular risk factors
Ribeiro [30]	2026	Comprehensive review	Patients with MASLD	Noninvasive fibrosis assessment	Reviewed the prognostic role of FIB-4, transient elastography, ELF score, and other fibrosis markers for cardiovascular risk stratification
Gbadamosi [31]	2025	Retrospective cohort	19,395 patients with MASH	FIB-4	High baseline and increasing FIB-4 scores independently predicted major adverse cardiovascular events and liver-related outcomes
Suresh [32]	2026	State-of-the-art review	Patients with MASLD	Fibrosis biomarkers	Summarized the evidence supporting FIB-4, NFS, ELF, and liver stiffness measurement as predictors of ASCVD independent of traditional cardiovascular risk factors
Oh [33]	2021	Prospective cohort (16-year follow-up)	Korean population	Liver fibrosis indices	Elevated liver fibrosis indices independently predicted long-term mortality and cardiovascular outcomes
Li [34]	2026	Comprehensive review	Patients with MASLD	ELF score	Higher ELF scores were associated with cardiovascular mortality and heart failure hospitalization, highlighting active fibrogenesis as a marker of systemic vascular injury
Sukocheva [35]	2025	Review	Patients with MASLD	Transient elastography	Demonstrated the consistent associations between liver stiffness measurements and increased cardiovascular risk, vascular stiffness, and coronary disease
Gries [36]	2025	Narrative/interdisciplinary review	Patients with MASLD, with emphasis on the MASLD-CAD relationship	Cardiovascular and vascular complications of MASLD and the potential causal relationship between MASLD and CAD	MASLD is strongly associated with cardiovascular disease, particularly CAD, with emerging evidence suggesting a potential causal relationship beyond shared metabolic risk factors
Lee [37]	2025	Nationwide cohort	134,686 patients with MASLD	FIB-4 and mortality	Higher FIB-4 independently predicted increased all-cause and cardiovascular mortality, particularly among younger individuals
Lee [38]	2025	Longitudinal cohort	Individuals with MASLD	Serial FIB-4 trajectories	Persistently elevated or increasing FIB-4 trajectories independently predicted progressive CAC accumulation
Tsai [39]	2022	Longitudinal imaging cohort	Patients with MASLD	Coronary plaque progression	Liver fibrosis biomarkers independently predicted coronary plaque progression, including among patients with low baseline coronary calcium scores
Lee [40]	2020	Prospective cohort	Patients with MASLD	Liver fibrosis and CAC	Advanced liver fibrosis independently predicted accelerated CAC progression
Ye [41]	2025	Cross-sectional study	Patients with MASLD	Carotid atherosclerosis	Increasing fibrosis severity enhanced the predictive value of Lp-PLA2 for carotid atherosclerosis
Jamalinia [42]	2024	Systematic review and meta-analysis	>12,000 patients with MASLD	Carotid atherosclerosis	Demonstrated a stepwise relationship between fibrosis severity and subclinical carotid atherosclerosis independent of conventional cardiovascular risk factors
Jeong [43]	2025	Observational cohort	Patients with MASLD	Coronary atherosclerosis	MASLD and fibrosis severity were independently associated with subclinical coronary atherosclerosis and high-risk plaque characteristics
Cernea [44]	2025	Cross-sectional study	Patients with type 2 diabetes and MASLD	Epicardial adipose tissue	Advanced fibrosis correlated with increased epicardial adipose tissue and an adverse cardiometabolic phenotype, supporting systemic cardiovascular involvement
Lee [45]	2025	Nationwide cohort	Patients with MASLD	Fibrosis severity and CAD	Increasing MASLD severity and hepatic fibrosis independently predicted incident CAD and higher overall cardiovascular risk after adjustment for traditional risk factors
Salem [46]	2025	Multicenter propensity-matched longitudinal cohort	48,552 patients with MASLD	Major adverse cardiovascular events	MASLD was associated with significantly increased risks of myocardial infarction, stroke, cardiovascular death, and overall MACE. Statin therapy significantly reduced cardiovascular risk
Brar [47]	2026	State-of-the-art review	Clinical and experimental evidence	Hepatic fibrosis and HFpEF	Reviewed mechanisms linking hepatic fibrosis with HFpEF through systemic inflammation, myocardial fibrosis, endothelial dysfunction, and ventricular remodeling
Nabilou [48]	2026	Cardiac magnetic resonance imaging study	Patients with advanced MASLD fibrosis	Myocardial remodeling	Advanced fibrosis was associated with increased myocardial extracellular volume and impaired diastolic strain, indicating diffuse myocardial fibrosis
Yang [49]	2026	Population-based cohort	Adults with MASLD	Incident atrial fibrillation	Greater liver fibrosis burden independently increased the risk of incident atrial fibrillation after adjustment for metabolic risk factors
Li [50]	2025	NHANES analysis	Young adults with MASLD	Arterial stiffness	MASLD was independently associated with increased arterial stiffness, supporting early vascular dysfunction even in younger populations
Zhang [51]	2025	Cross-sectional study	481 patients with MASLD	Lower extremity arterial disease	Higher FIB-4 categories independently predicted lower extremity arterial disease, extending the vascular manifestations of hepatic fibrosis beyond coronary disease
Zhang [52]	2025	Cardiovascular mortality analysis	Patients with MASLD	Fibrosis-integrated inflammatory markers	Combining FIB-4 with systemic inflammatory indices significantly improved the prediction of cardiovascular mortality compared with fibrosis assessment alone
Ros-Madrid [53]	2025	Comprehensive review	Patients with MASLD	GLP-1 receptor agonists	Reviewed the anti-fibrotic, anti-inflammatory, metabolic, and cardioprotective effects of GLP-1 receptor agonists in MASLD
Newsome [54]	2021	Phase 2 randomized controlled trial	320 patients with biopsy-confirmed MASH	Semaglutide	Semaglutide significantly increased MASH resolution without worsening fibrosis while improving cardiometabolic risk factors
Sanyal [55]	2025	Phase 3 randomized clinical trial	Patients with MASH	Semaglutide	Demonstrated clinically meaningful reductions in liver stiffness, body weight, inflammatory biomarkers, and atherogenic lipid parameters, suggesting potential cardiovascular benefit
Loomba [56]	2024	SYNERGY-NASH phase 2 randomized trial	Patients with biopsy-confirmed MASH	Tirzepatide	Tirzepatide achieved high rates of MASH resolution and fibrosis improvement with parallel reductions in ApoB, triglycerides, and systemic inflammation
Sattar [57]	2021	Systematic review	Patients with type 2 diabetes	GLP-1 receptor agonists	Demonstrated significant reductions in myocardial infarction, stroke, and cardiovascular mortality with GLP-1 receptor agonist therapy
Kuchay [58]	2018	Randomized controlled trial	Patients with type 2 diabetes and NAFLD	Empagliflozin	Empagliflozin significantly reduced liver fat content compared with standard therapy and improved metabolic parameters, supporting its potential role in improving hepatic steatosis and cardiometabolic health
Erfanifar [59]	2025	Randomized clinical trial	Patients with type 2 diabetes and NAFLD	Empagliflozin	Empagliflozin significantly reduced liver fat content compared with standard therapy and improved metabolic parameters, supporting its potential role in improving hepatic steatosis and cardiometabolic health
Eriksson [60]	2018	Randomized clinical trial	Patients with MASLD and type 2 diabetes	Dapagliflozin	Dapagliflozin reduced epicardial adipose tissue volume and improved coronary flow reserve, supporting direct vascular benefits
McGuire [61]	2021	Systematic review and meta-analysis of cardiovascular outcome trials	Patients with diabetes and heart failure	SGLT2 inhibitors	Demonstrated consistent reductions in hospitalization for heart failure and cardiovascular mortality across diverse high-risk populations
Harrison [62]	2024	Phase 3 clinical trial	Patients with MASH	Resmetirom	Resmetirom significantly improved liver fibrosis while reducing LDL cholesterol, ApoB, and triglyceride concentrations, suggesting dual hepatic and cardiovascular benefit
Lurje [63]	2024	Review	Patients with MASH	MAESTRO-NASH trial	Reviewed evidence supporting resmetirom as the first approved anti-fibrotic therapy for MASH with additional cardiometabolic benefits
Francque [64]	2021	NATIVE phase 2 randomized trial	Patients with MASH	Lanifibranor	Lanifibranor significantly improved fibrosis stage and inflammatory activity while improving metabolic dysfunction
Sanyal [65]	2024	Phase 2 clinical trial	Patients with MASH	Survodutide	Survodutide reduced liver fat content, fibrosis biomarkers, body weight, and inflammatory markers associated with vascular dysfunction
Browne [66]	2025	Phase 2 clinical trial	Patients with MASH	Pemvidutide	Pemvidutide significantly reduced hepatic fat fraction and improved triglycerides, hsCRP, and systemic inflammatory burden, supporting integrated cardio-hepatic benefits
Jastreboff [67]	2023	Phase 2 randomized clinical trial	Adults with obesity	Retatrutide	Triple GLP-1/GIP/glucagon receptor agonism produced profound weight loss with improvements in insulin resistance and cardiometabolic risk factors that may indirectly reduce hepatic fibrosis and ASCVD risk
Vilar-Gomez [68]	2015	Prospective cohort with serial liver biopsies	Patients with biopsy-proven NASH	Weight loss and fibrosis regression	Sustained weight loss ≥10% resulted in fibrosis regression and resolution of steatohepatitis in a substantial proportion of patients
Keating [69]	2012	Randomized controlled trial	Adults with NAFLD	Aerobic exercise	Exercise reduced hepatic fat, improved endothelial function and arterial stiffness, and enhanced vascular health independent of significant weight loss
Estruch [70]	2018	Randomized clinical trial (PREDIMED)	High cardiovascular risk population	Mediterranean diet	Mediterranean dietary intervention significantly reduced cardiovascular events while improving metabolic and inflammatory profiles relevant to MASLD
Gepner [71]	2018	Randomized controlled trial	Adults with abdominal obesity/dyslipidemia	Mediterranean diet	Green Mediterranean diet produced greater reductions in hepatic fat content and improved cardiometabolic risk markers compared with standard healthy dietary advice
Hallsworth [72]	2011	Randomized controlled trial	Patients with NAFLD	Resistance exercise	Resistance exercise reduced hepatic steatosis and improved insulin sensitivity and vascular function even without major weight reduction
Visseren [73]	2021	European Society of Cardiology Clinical Practice Guideline	Adults at cardiovascular risk	Cardiovascular prevention	Updated the cardiovascular prevention guidelines emphasizing comprehensive risk assessment and aggressive management of cardiometabolic risk factors
Lu [74]	2025	Prospective cohort	Patients with type 2 diabetes	Hepatic fibrosis and ASCVD risk	Metabolic risk factors significantly modified the association between hepatic fibrosis and atherosclerotic cardiovascular risk, supporting integrated liver-cardiometabolic risk assessment
Boeckmans [75]	2025	Prospective cohort	Patients with heart failure with or without MASLD	FIB-4 mortality	The FIB-4 index independently predicted all-cause mortality in heart failure patients regardless of MASLD status, highlighting its prognostic cardiovascular value
Huang [76]	2024	NHANES cohort analysis	Adults with MASLD	Liver stiffness versus steatosis	Liver stiffness, rather than steatosis alone, independently predicted mortality, emphasizing fibrosis as the major prognostic determinant
Crespo [77]	2026	Narrative review	Patients with MASLD	Cardiovascular risk	Reviewed the bidirectional relationship between MASLD and cardiovascular disease and emphasized multidisciplinary cardiovascular risk management
Romeo [78]	2008	Genetic association study	Patients with fatty liver disease	PNPLA3 polymorphism	Identified PNPLA3 as a major genetic determinant of hepatic steatosis and fibrosis susceptibility
Dongiovanni [79]	2015	Genetic association study	MASLD populations	TM6SF2 polymorphism	Demonstrated that the TM6SF2 variant promotes hepatic fat accumulation and fibrosis while exerting complex effects on cardiovascular risk
Babu [80]	2024	Metabolomic cohort study	Severely obese patients with MASLD	Metabolomic signatures	Identified the metabolomic profiles associated with MASLD severity that may contribute to cardiometabolic dysfunction
Zhou [81]	2025	Narrative review	39 empirical CAD risk-prediction studies	Multimodal data fusion for precision CAD risk prediction	Integrating imaging, clinical, genomic, EHR, and wearable data improves CAD risk discrimination; deep/graph neural networks enable multimodal prediction, but heterogeneity, bias, limited external validation, and workflow integration remain key barriers
Agarbattiwala [82]	2025	Retrospective cohort study	2,000 adults aged 30-75 years; RF, SVM, and DNN models	AI versus conventional FRS and ASCVD risk scores for cardiovascular event prediction	DNN showed superior predictive performance (AUC 0.91) compared with RF (0.87), SVM (0.84), FRS (0.76), and ASCVD (0.74), supporting the potential of AI for improved cardiovascular risk stratification
Fouad [83]	2020	Expert review	Patients with fatty liver disease	Disease nomenclature	Discussed the rationale and implications of transitioning from NAFLD to MAFLD terminology
Lazarus [84]	2022	Global policy review	International health systems	NAFLD policy preparedness	Evaluated the worldwide preparedness for fatty liver disease management and emphasized the need for coordinated public health strategies
Moolla [85]	2019	Prospective multidisciplinary clinic study	Patients with NAFLD	Multidisciplinary management	Demonstrated that multidisciplinary care significantly improved liver-related and cardiometabolic outcomes
Wattacheril [86]	2018	Review	NASH	Emerging anti-fibrotic therapies	Summarized the novel pharmacologic therapies targeting hepatic fibrosis and steatohepatitis
Byrne [87]	2020	Review	NAFLD	Extrahepatic complications	Reviewed the mechanistic relationship between NAFLD and chronic kidney disease, highlighting the systemic consequences of hepatic fibrosis
Younossi [88]	2019	Phase 3 randomized clinical trial	Patients with NASH	Obeticholic acid	Interim phase 3 results demonstrated improvement in liver fibrosis with obeticholic acid compared with placebo
Malandris [89]	2026	Comprehensive review	Patients with MASLD and type 2 diabetes	Pharmacologic therapy	Reviewed the current and emerging pharmacologic treatments for MASLD in the context of type 2 diabetes, emphasizing therapies with both hepatic and cardiometabolic benefits
